# Self-expandable metal stent of esophagogastric junction versus pyloric area obstruction in advanced gastric cancer patients: Retrospective, comparative, single-center study

**DOI:** 10.1097/MD.0000000000021621

**Published:** 2020-08-14

**Authors:** Deok Yeong Kim, Hee Seok Moon, In Sun Kwon, Jae Ho Park, Ju Seok Kim, Sun Hyung Kang, Eaum Seok Lee, Seok Hyun Kim, Byung Seok Lee, Jae Kyu Sung, Hyun Yong Jeong

**Affiliations:** a Division of Gastroenterology, Department of Internal Medicine, Daejeon Veterans Hospital; b Division of Gastroenterology, Department of Internal Medicine, Chungnam National University Hospital; c Clinical Trials Center, Chungnam National University Hospital, Daejeon, South Korea.

**Keywords:** gastric cancer, self-expandable metal stent, gastric malignant obstruction, palliative, esophagogastric junction

## Abstract

Upper gastrointestinal stenting is a palliative treatment for relieving symptoms such as nausea, vomiting, and dietary intake in patients with obstruction due to inoperable advanced stomach cancer. Self-expandable metal stent (SEMS) implantation for malignant obstruction has recently become more effective, safer, and less expensive than operative modality. It also has better short-term outcomes, particularly a shorter hospital stay and a more rapid return to oral intake, than surgical treatment. However, there is no comparative analysis regarding the efficacy, side effects, and survival rate of stenting between the esophagogastric junction (EGJ) and pyloric obstructions.

To compare the prognoses and complications after SEMS implantation between EGJ and pyloric obstructions in advanced gastric cancer.

Among advanced gastric cancer patients with gastrointestinal obstruction diagnosed from January 2008 to December 2017 at the Gastroenterology Department of Chungnam National University Hospital, 42 and 76 patients presented with EGJ (EGJ obstruction group) and gastric pyloric obstructions (pyloric obstruction group), respectively. We retrospectively reviewed the survival period, changes in food intake, and complications of these patients before and after SEMS placement.

The prevalences of aspiration pneumonia were 11.9% (5/42) and 2.6% (2/76) in the EGJ and pyloric obstruction groups, respectively, before SEMS placement (*P* value: .041). Other symptoms associated with gastric malignant obstruction were not statistically different between the groups. Success rate and adverse events did not significantly differ between the EGJ and pyloric obstruction groups. There was no difference in frequency of stent reinsertion procedures performed owing to reobstruction, but the reprocedure average period was statistically significantly longer in the EGJ obstruction group [EGJ obstruction: 158.3 days (±42.4); pyloric obstruction: 86.0 days (±29.1)] (*P* value: .022). As an index of improved dietary status, the Gastric Outlet Obstruction Scoring System score was not significantly different between the groups before and after SEMS placement.

The EGJ and pyloric obstruction groups did not significantly differ in prognosis or complication rates. However, EGJ stent was more stable than pyloric stent when reobstruction was considered.

## 1 Introduction

Gastric cancer is the second leading cause of cancer-related death worldwide^[1]^. Malignant gastric obstruction and related symptoms induced by the progression of advanced gastric cancer have a significant impact on quality of life.^[2–7]^

Currently, self-expandable metal stent (SEMS) implantation of the upper gastrointestinal tract is a palliative treatment for the relief of gastrointestinal obstructive symptoms, such as nausea, vomiting, regurgitation, poor oral intake, and malnutrition, in patients with gastrointestinal obstruction caused by growth of malignant tumors.^[8]^ To improve these symptoms, various treatment options, such as surgery, SEMS implantation, and palliative radiation therapy, can be considered. In the past, the standard minimally invasive treatment for malignant pyloric obstruction was gastrojejunostomy (GJ). However, endoscopic gastroduodenal SEMS implantation for malignant pyloric stricture recently has become more effective, safer, and less expensive than GJ. It also has better short-term outcomes than GJ, particularly a shorter hospital stay and a more rapid return to oral intake.^[9,10]^

The obstruction due to advanced gastric cancer is anatomically classified as the esophagogastric junction (EGJ) area and pyloric area, and stent insertion is frequently performed at these sites. Despite many studies, however, there is no comparative analysis on the efficacy, side effects, and survival rate of stenting between the EGJ and pyloric obstructions. The purpose of this article is to compare the prognosis and complications between EGJ SEMS and pyloric SEMS used for malignant obstructions in patients with advanced gastric cancer.

## 2 Materials and methods

### 2.1 Patients and study design

**Figure 1:**
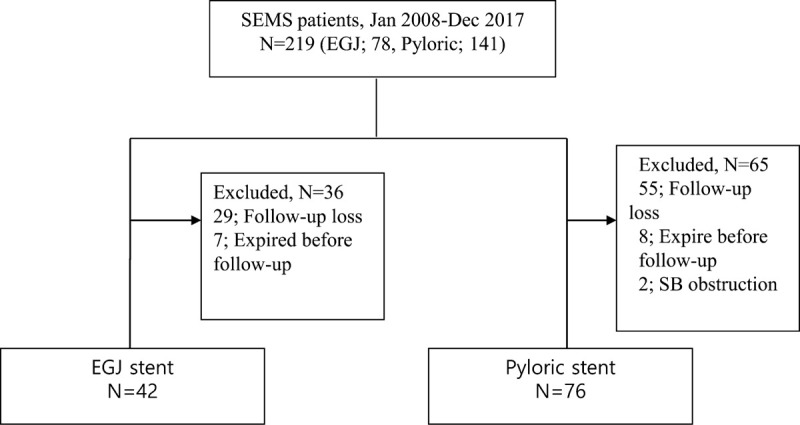
Flow diagram of patients enrolled in this study. The 42 patients in the EGJ obstruction group and the 76 patients in the pyloric obstruction group were selected by applying the study's inclusion and exclusion criteria. A total of 118 patients were enrolled. EGJ = esophagogastric junction, SB = small bowel, SEMS = self-expandable metal stent.

All consecutive patients who underwent SEMS implantation for EGJ and pyloric obstructions due to advanced gastric cancer between January 2008 and December 2017 at the department of gastroenterology of Chungnam National University Hospital were identified by searching the medical databases of the hospital. Patients were divided into the following groups: EGJ obstruction group consisting of 42 patients who showed gastric obstruction of the EGJ and pyloric obstruction group comprising 76 patients who had pyloric area obstruction among patients with gastrointestinal obstruction diagnosed by radiological or endoscopic examinations (Fig. 1). At 3 months after the procedure, changes in food intake, complications, and nutritional parameters were retrospectively reviewed.

All patients were pathologically diagnosed with adenocarcinoma by endoscopic biopsy. Imaging studies such as computed tomography were performed to determine the progressive stage of the lymph node or other organ metastasis. TNM staging system was based on the recently revised American Joint Committee on Cancer (AJCC) 8th edition. Patients were mostly unsuitable to undergo surgical therapy due to their systemic condition or presence of tumor metastasis. The EGJ obstruction group was limited to patients in whom the main lesion located in the cardia area and with some esophageal invasions, whereas the pyloric obstruction group was limited to those in whom the main lesion was located in the antrum and with invasion to the p-ring or duodenal bulb.

Patients were included in the study if they had undergone endoscopic SEMS for EGJ or pyloric obstruction, they had documented, unresectable gastric adenocarcinoma, and they had obstructive symptom of the EGJ or pyloric area that was causing nausea, vomiting, dysphagia, and oral intake difficulties. Patients were excluded if they were lost to follow-up during a specified period; they expired before the follow-up period; gastric malignant obstruction was caused by esophageal cancer or duodenal cancer, or extragastric obstruction was caused by an external tumor compression; and intestinal enterography with contrast medium revealed multiple small bowel obstructions.^[11]^

This study was reviewed and approved by the Chungnam National University Hospital Institutional Review Board (IRB file No. CNUH 2019-02-020). And this study is a retrospective study using medical records, and personal information protection measures are appropriately established so that the informed consent of the subject can be exempted.

### 2.2 Definitions

The outcome of SEMS implantation was evaluated according to the following parameters: technical success rate, clinical success rate, and duration of stent patency.

Technical success rate was defined as the successful insertion of a stent in the correct position and the confirmation of patency by endoscopic and fluoroscopic examinations with oral contrast opacification. Clinical success rate was defined as an improvement in the obstructive symptoms and oral intake 1 to 3 days after stent placement.

The degree of oral intake was assessed using the Gastric Outlet Obstruction Scoring System (GOOSS), which was as follows: no oral intake (0), exclusively liquid diet (1), exclusively soft solid diet (2), and full diet was possible (3).^[12]^ The improvement in oral intake was evaluated as the best degree at least 3 days after stent insertion. Primary stent dysfunction was defined as a failure to resume an oral diet after pyloric stenting.

Performance status was assessed using the Eastern Cooperative Oncology Group (ECOG) scale, with the following rating: 0, normal activity; 1, with symptoms but ambulatory; 2, in bed 50% or less of the time; 3, in bed >50% of the time; and 4, totally bedridden^[12]^. ECOG status was prospectively recorded and then retrospectively reviewed.

Duration of stent patency was defined as the period between the initial stent placement and the recurrence of obstructive symptoms caused by tumor in-growth.^[12,13]^

### 2.3 Materials and placement of SEMS

Endoscopic stent insertion was performed using Hanaro stents (M.I. Tech Co Ltd, Seoul, Korea) in both groups. In the EGJ obstruction group, stent size mainly used had a diameter of 20 to 22 mm and a length of 9 to 14 cm depending on the case. In the pyloric group, stent size mainly used had a diameter of 18 to 20 mm and a length of 6 to 12 cm depending on the case. Uncovered stent types were mainly used in both groups. Main endoscope bodies used were CV-260 or CV-290 (Olympus Co., Japan), and the endoscope fiber used had a dual-channel endoscope (GIF-2T240, Olympus Co) with an internal diameter of 3.7 and 2.8 mm, respectively.

**Figure 2:**
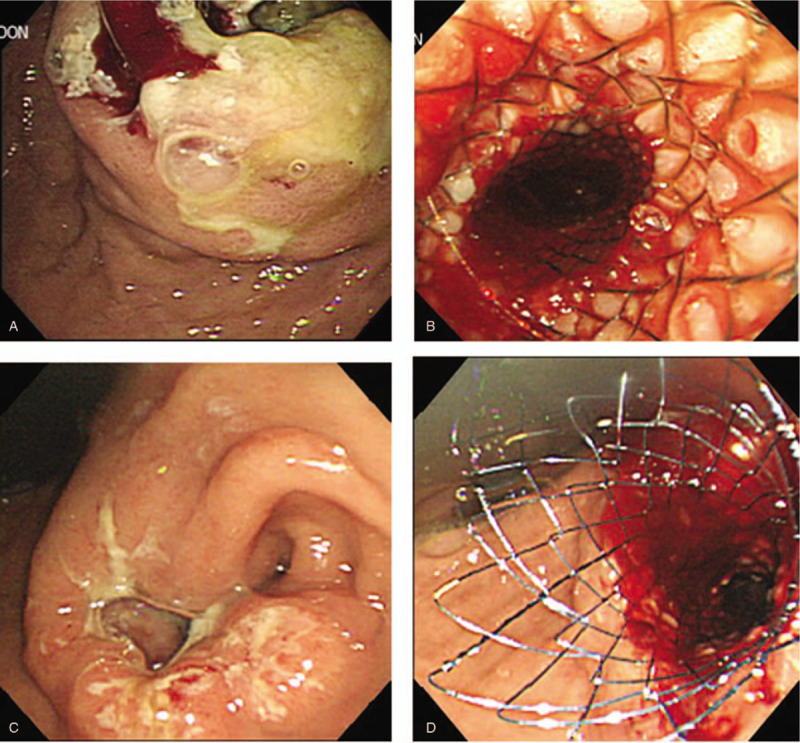
Placement of the SEMS in both groups. A, Obstructed EGJ lumen before SEMS insertion. B, EGJ with extended lumen after SEMS insertion. C, Obstructed pyloric area before SEMS insertion. D, Pyloric area with extended lumen after SEMS insertion. EGJ = esophagogastric junction, SEMS = self-expandable metal stent.

SEMS placement was performed after confirming the location and length of the gastric obstruction under upper gastrointestinal fluoroscopic guidance. The patient was placed on a decubitus position and pretreated with midazolam, followed by upper gastrointestinal endoscopy. At the endoscopic view, radiographic examination was performed in parallel, and the obstruction was confirmed. A guidewire was inserted using a standard catheter. The length of the stent was allowed to remain at least 1 to 2 cm in the proximal and distal portions of the obstruction. SEMS was inserted to the distal portion of the obstruction through the endoscopic forceps, and the dilatation was initiated from the distal portion of the obstruction site. During stenting, the stent tended to displace more distally than expected; thus, the stent was deployed while adjusting the proximal position. After expanding SEMS, the resolution of obstruction was confirmed by endoscopic and contrast injection, and complications such as bleeding, dislocation, and perforation were observed (Fig. 2).

### 2.4 Statistical analysis

Baseline clinical characteristics are expressed as the number (percentage) for categorical variables or the mean ± standard deviation for continuous variables. Categorical variables were compared using Fisher exact test or chi-squared test, and continuous variables were compared using Student *t* test. Overall survival curves were determined by using the Kaplan-Meier method and compared with the log-rank test. Univariate and multivariate analyzes were performed to predict changes in the index after the onset of the meal. All *P* values were bilateral, and *P* < .05 was considered statistically significant. All statistical analyses were performed using Statistical Package for the Social Sciences version 21.0 (IBM Co., Armonk, NY).

## 3 Results

### 3.1 Baseline characteristics between the 2 groups

The mean age of the EGJ and pyloric obstruction groups was 67.7 and 64.6 years, respectively, and the male ratio was 88.1% and 80.3%, respectively. Stage IV tumors were most common in both groups [36 (85.7%) in the EGJ obstruction group and 66 (86.8%) in the pyloric obstruction group]. The most common site of metastasis was liver in both groups. The average ECOG scores were 2.32 and 1.95 in both the EGJ and pyloric obstruction groups, respectively. Chemotherapy administration had similar performance rates in both groups. A measure of the degree of improvement in dietary status, the GOOSS scores before treatment were not significantly different between the 2 groups (Table 1).

**Table 1:**
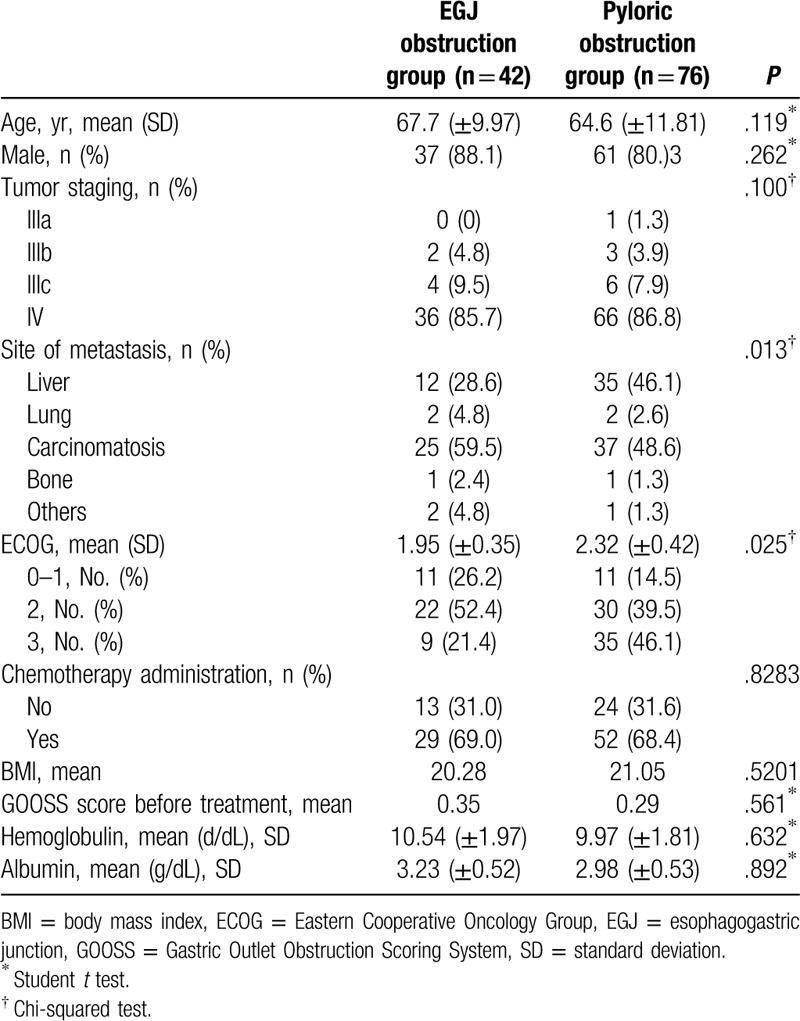
Baseline characteristics between the esophagogastric junction and pyloric obstruction groups.

### 3.2 Associated symptom between the 2 groups before stent insertion

Regarding the associated symptoms, weight loss or anorexia was observed in 60.9% (29/42) and 82.9% (63/76) of the patients in the EGJ and pyloric obstruction groups, respectively (*P* value: .082), whereas nausea or indigestion were observed in 92.9% (39/42) of the patients in the EGJ obstruction group, which was higher compared to the 81.6% (62/76) in the pyloric obstruction group (*P* value: .095). However, the difference was not significant between the 2 groups. However, when comparing the incidence of aspiration pneumonia before stent insertion, the EGJ obstruction group had higher incidence than the pyloric obstruction group [11.9% (5/42) vs 2.6% (2/76)]. This was the only statistically significant data among the symptoms caused by gastric malignant obstruction before stent insertion (*P* value: .041) (Table 2).

**Table 2:**
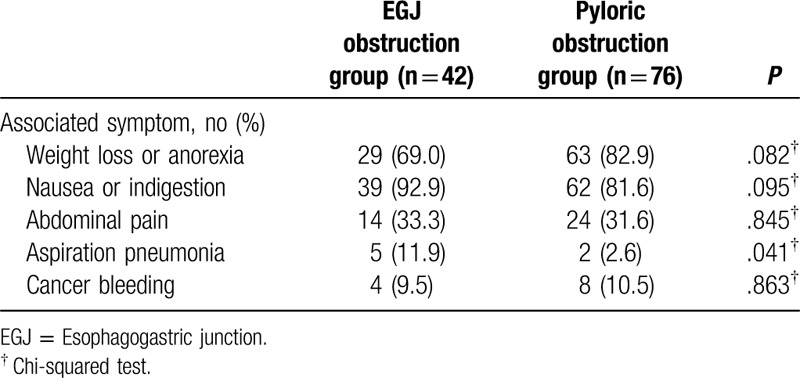
Comparison of associated symptom between the esophagogastric junction and pyloric obstruction groups before stent insertion.

### 3.3 Procedure success rates and adverse events between the 2 groups

Success rates compared were clinical and technical success rates. In both groups, the clinical and technical success rates were >80%, with the pyloric obstruction group showing higher success rates. However, there was no statistical difference in the success rate between the groups (Table 3).

**Table 3:**
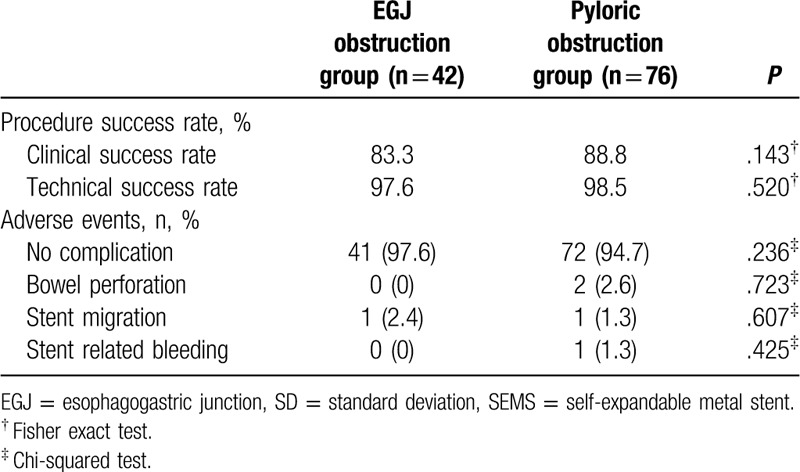
Comparison of procedure success rate and adverse events between the esophagogastric junction and pyloric obstruction groups.

Adverse events after the procedure were not >90% in both groups. When comparing the adverse events by type, there was no significant difference in the incidence and number of dislocation and stent-related bleeding between the 2 groups. Moreover, perforation, one of the important complications, was observed in 2 patients in the pyloric obstruction group and none in the EGJ obstruction group, but this difference was not significant when considering the statistical value, but it is relatively low compared to those of other studies^[14]^ (Table 3).

### 3.4 Length and diameter of SEMS and duration of stent patency between 2 groups

The lengths of the procedure stents were 11.5 and 10.6 cm, respectively; the EGJ obstruction group had a slightly longer stent. In terms of stent diameter, both groups were inserted with stents with approximately 20 mm in diameter. No significant differences in length and diameter of SEMS were found between the groups.

**Figure 3:**
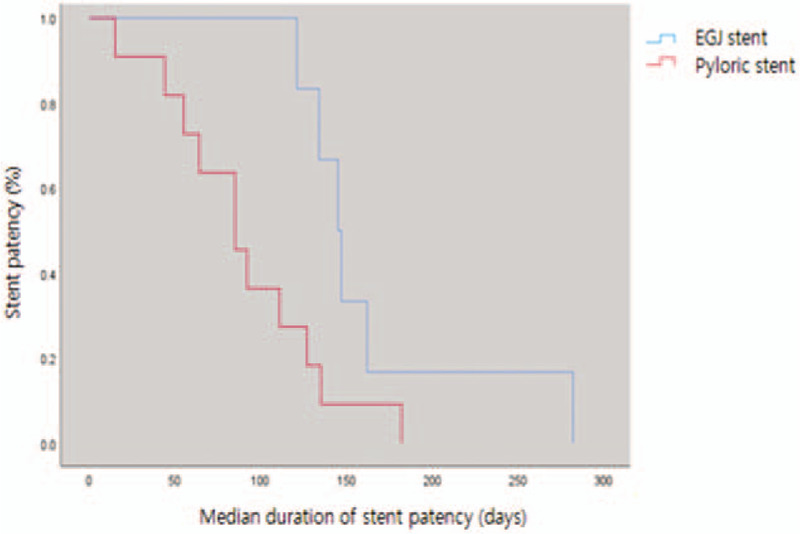
The Kaplan-Meier curve of the duration of stent patency between the EGJ and pyloric obstruction groups. EGJ = esophagogastric junction.

The difference in reprocedure was 16.7% (7/42) in the EGJ obstruction group, whereas 13.2% (10/76) in the pyloric obstruction group (*P* value = .901). In the results of the comparative analysis of tumor in-growth or stent patency that causes reprocedure (Table 5), tumor in-growth rates were >10% in both groups. The EGJ obstruction group had a higher tumor in-growth rate compared to the pyloric obstruction group (11.9% vs 14.5%) (*P* value: .723). When comparing the *duration of stent patency*, the average *duration* of the EGJ obstruction group was 158.3 days, whereas that of the pyloric obstruction group was 86.0 days, which showed statistically significant difference (*P* value: .022) (Fig. 3). This suggests that the EGJ stent remains more stable after insertion (Table 4).

**Table 4:**
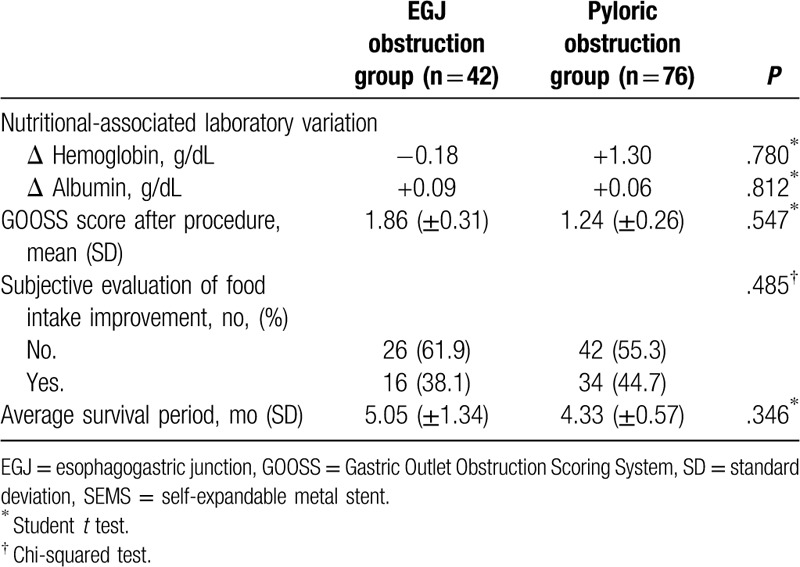
Comparison of nutritional effect and survival period between the esophagogastric junction and pyloric obstruction groups.

### 3.5 Nutritional effect and survival period between the 2 groups

**Figure 4:**
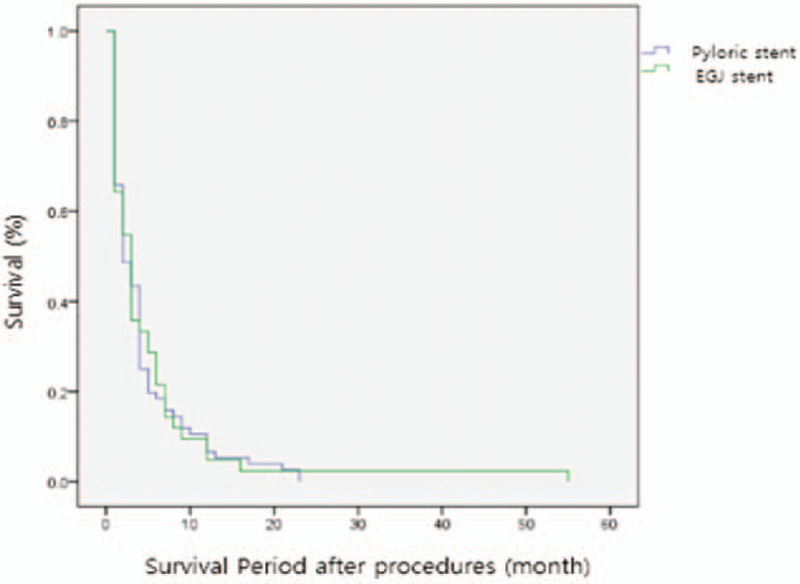
The Kaplan-Meier curve of the survival rates between the EGJ and pyloric obstruction groups. EGJ = esophagogastric junction.

When the laboratory changes before and after the insertion of the stent were investigated, variation in hemoglobin and albumin levels was not statistically different in both groups. The improvement rate of food intake after the procedure was 61.9% (EGJ obstruction group) and 55.3% (pyloric obstruction group) (*P* value: .485). Moreover, the GOOSS score was also increased in both groups, without statistical significance (*P* value: .547). The difference in survival periods between the 2 groups was 5.05 and 4.33 months in the EGJ and pyloric obstruction groups, respectively. Although the EGJ obstruction group had a longer survival period (by 0.7 months), statistical significance was not confirmed (Table 5, Fig. 4).

**Table 5:**
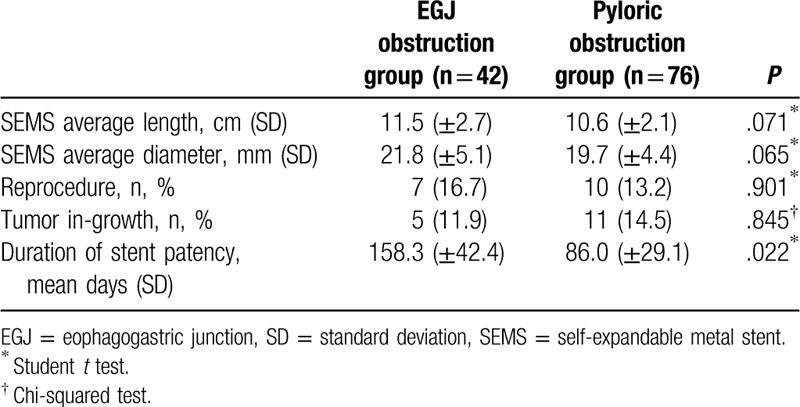
Comparison of length and diameter of self-expandable metal stent and tumor in-growth related to duration of stent patency between the esophagogastric junction and pyloric obstruction groups.

## 4 Discussion

SEMS implantation is a safe and effective procedure for palliation and is currently the mainstay nonsurgical modality in patients with malignant obstruction^[12,15,16]^. Definitely, palliative radiotherapy may be discussed as an option for treatment; however, in the present study, we compared the effects of physical improvement of SEMS implantation, rather than slow response radiotherapy in terms of improving the quality of life in the acute phase where dietary intake is not possible due to gastric obstruction.

In this study, we compared the efficacy, survival periods, and adverse effects of the EGJ and pyloric obstruction groups. Regarding the associated symptoms before the procedure, aspiration pneumonia occurred more frequently in the EGJ obstruction group [5/42 cases (11.9%) in EGJ obstruction group vs 2/76 cases (2.6%) in the pyloric obstruction group, *P* = .041]. The higher incidence of aspiration pneumonia in the EGJ obstruction group appears to be due to anatomical causes.^[17]^ Compared to patients with pyloric obstruction, it is thought that patients with EGJ obstruction are more likely to have aspiration owing to food regurgitation due to the higher food retention. However, there were no significant differences in other associated symptoms, and there was no difference in survival rates and treatment efficacy between the 2 groups.

In terms of clinical and technical success rates, technical success rates were >95% in both groups (97.6% in the EGJ obstruction group vs 98.5% in the pyloric obstruction group, *P* = .520). Previous studies using endoscopic guidance have reported high technical feasibility rates of 93.5% to 100%.^[13,18]^ Clinical success rates of this study were all less than 90% in both groups (83.8% in the EGJ obstruction group vs 88.8% in the pyloric obstruction group, *P* = .143), which are similar to those of previous studies reporting on symptomatic improvement in 70% to 100% of cases.^[19]^ Considering the lack of statistical significance, the lesion characteristics had no influence on the procedure success rate of both groups.^[20]^

The EGJ group had longer stent length of about 0.9 cm than the pyloric obstruction group (11.5 ± 2.7 vs 10.6 ± 2.1 cm *P* = .071). Moreover, the EGJ obstruction group had stent diameters larger by 2.1 mm than the pyloric obstruction group (21.8 ± 5.1 vs 19.7 ± 4.4 mm *P* = .065). Considering the anatomical differences, the difference in length and diameter between the 2 groups was not significant. This difference seems to be due to the use of standardized instruments in both groups. The other studies have noted that the analyzed factors affecting these applications include the type of stent, as well as the presence of chemotherapy and radiation therapy.^[21–23]^ However, our study used a similar type of uncovered stent and patients received a similar rate of chemotherapy, indicating that our population was more homogeneous compared to other studies.

The adverse events were also higher in the pyloric obstruction group than in the EGJ obstruction group, but showing no significant difference. Two cases of bowel perforation were detected in a patient who had a 10-cm uncovered stent inserted at the pyloric area. Stent migration was found in 1 patient in each group (*P* = .607), and 1 patient in the pyloric obstruction group had stent-related bleeding (1.3%, *P* = .425).

Tumor in-growth, which is an important cause of reprocedure, was not statistically significant in both groups [5 of 42 cases (11.9%) in the EGJ obstruction group vs 11 of 76 cases (14.5%) in pyloric obstruction group, *P* = .845]. However, the duration of stent patency was different in both groups. It was approximately 72.3 days longer in the EGJ obstruction group (158.3 ± 42.4 vs 86.0 ± 29.1 days, *P* = .022). This suggests that the EGJ stent is relatively more stable than the pyloric stent. Considering that tumor in-growth is not statistically significant, 2 assumptions can be made about this. First, it may be that the pyloric area has more severe peristalsis for bowel movements and food impaction caused by food congestion for a longer time than the EGJ area.

A previous study reported an improvement of GOOSS score, which shows the improvement of the patients’ physical dietary intake, of approximately 80%.^[13]^ In this study, the GOOSS score showed a similar improvement of 78%. When comparing the GOOSS score before and after the procedure, both groups showed an increase of approximately 1.5 points in both groups (1.86 ± 0.31 in the EGJ obstruction group vs 1.24 ± 0.26 in the pyloric obstruction group, *P* = .547). Although there is no statistical significance, both procedures have an influence on the improvement of dietary status.

Hemoglobin and albumin levels were also identified as indicators of dietary improvement. There was no significant difference in the hemoglobin levels between the 2 groups (−0.18 g/dL in the EGJ obstruction group vs +1.30 g/dL in the pyloric obstruction group, *P* = .780). In this analysis, hemoglobin was thought to have been influenced by transfusion, blood loss due to cancer bleeding, and iron deficiency anemia frequently observed in patients with stomach cancer.^[24]^ Although the albumin level has risen to a very small extent (−0.18 g/dL in the EGJ obstruction group vs +1.30 g/dL in the obstruction group, *P* = .812), there is a limit to the fact that a relatively poor nutritional status and a long follow-up period are required to raise the albumin characteristics.^[25,26]^

The effectiveness of the procedure in the actual clinical practice is thought to have decreased with respect to the condition and progression of the patient's tumor. The survival time of the patients was 5.05 ± 1.34 and 4.33 ± 0.57 months in the EGJ and pyloric observation groups, respectively (*P* = .346), but there was no significant difference observe in reference to the Kaplan-Meier curve. This was probably due to the small sample size in both groups and the individual differences in tumor progression.

Our study has some limitations. First, it had a small sample size, was not randomized, was retrospective in nature, and patients were from a single center. Second, many of the relevant symptoms and dietary status assessments relied on the patient's subjective opinion. The resulting value would have caused some errors. Third, our study may have some errors in recruiting patients with anatomical location of stenting. In the EGJ obstruction group, it is probable that some cases of esophageal adenocarcinoma according to the latest 8th edition of TNM staging were included, and in the pyloric obstruction group, cases of borderline tumors such as duodenal cancer may have been included. Fourth, in the evaluation of the survival period after the procedure, it was thought that there was an effect of tumor progression rather than dietary status of the patient. Fifth, the selective reporting of studies with positive results may result in overestimation of the technical and clinical success rate as well as stent patency, resulting in some biases to our meta-analysis.

In conclusion, the prevalence of aspiration pneumonia was higher in the EGJ obstruction group than in the pyloric obstruction group, and, in terms of stability of the SEMS, the EGJ obstruction group showed superior results than the pyloric obstruction group. However, no other important differences in efficacy, side effects, and prognosis were found between the 2 groups. For more meaningful results, large-scale studies involving larger data will be needed in the future.

## Acknowledgments

HY Jeong, SH Kang, and JK Sung helped with data interpretation that was used in the current study; SH Kim, ES Lee, and BS Lee provided input and organized the data for statistical analysis; JH Park and JS Kim helped with data analysis; IS Kwon provided advice for the statistical analysis; all of the authors approved the final version of the manuscript.

## Author contributions

**Conceptualization:** Deok Yeong Kim.

**Data curation:** Deok Yeong Kim, In Sun Kwon.

**Formal analysis:** Deok Yeong Kim, Hee Seok Moon, Jae Ho Park, Ju Seok Kim, Eaum Seok Lee, Seok Hyun Kim, Byung Seok Lee, Jae Kyu Sung, Hyun Yong Jeong.

**Investigation:** Deok Yeong Kim, Sun Hyung Kang, Eaum Seok Lee, Seok Hyun Kim, Byung Seok Lee, Jae Kyu Sung, Hyun Yong Jeong.

**Methodology:** Deok Yeong Kim, Hee Seok Moon.

**Project administration:** Deok Yeong Kim, Hee Seok Moon.

**Resources:** Deok Yeong Kim.

**Software:** Deok Yeong Kim, In Sun Kwon.

**Supervision:** Hee Seok Moon.

**Validation:** Deok Yeong Kim, Hee Seok Moon.

**Writing – original draft:** Deok Yeong Kim.

**Writing – review & editing:** Deok Yeong Kim.
